# RNA-seq transcriptome analysis of breast cancer cell lines under shikonin treatment

**DOI:** 10.1038/s41598-018-21065-x

**Published:** 2018-02-08

**Authors:** Kuo-Hua Lin, Ming-Yii Huang, Wei-Chung Cheng, Shu-Chi Wang, Shih-Hua Fang, Hung-Pin Tu, Chia-Cheng Su, Yung-Li Hung, Po-Len Liu, Chi-Shuo Chen, Yu-Ting Wang, Chia-Yang Li

**Affiliations:** 1 Department of Surgery, Changhua Christian Hospital, Changhua City, 50006 Taiwan; 2 Department of Radiation Oncology, Cancer Center, Kaohsiung Medical University Hospital, Kaohsiung Medical University, Kaohsiung, 80708 Taiwan; 3 Graduate Institute of Biomedical Sciences, China Medical University, Taichung, 40402 Taiwan; 4 Health Management Center, Kaohsiung Medical University Hospital, Kaohsiung Medical University, Kaohsiung, 80708 Taiwan; 5 Institute of Athletics, National Taiwan University of Sport, Taichung, 40404 Taiwan; 6 Department of Public Health and Environmental Medicine, School of Medicine, College of Medicine, Kaohsiung Medical University, Kaohsiung, 80708 Taiwan; 7 Division of Urology, Department of Surgery, Chi-Mei Medical Center, Tainan, 71004 Taiwan; 8 Graduate Institute of Medicine, College of Medicine, Kaohsiung Medical University, Kaohsiung, 80708 Taiwan; 9 Department of Senior Citizen Service Management, Chia Nan University of Pharmacy and Science, Tainan, 71710 Taiwan; 10 Graduate School of Sport Sciences, Waseda University, Tokorozawa, 359-1192 Japan; 11 Department of Respiratory Therapy, College of Medicine, Kaohsiung Medical University, Kaohsiung, 80708 Taiwan; 12 Department of Biomedical Engineering and Environmental Sciences, National Tsing Hua University, Hsinchu, 30013 Taiwan; 13 Center for Infectious Disease and Cancer Research, Kaohsiung Medical University, Kaohsiung, 80708 Taiwan; 14 Department of Medical Research, Kaohsiung Medical University Hospital, Kaohsiung, Kaohsiung, 80756 Taiwan

## Abstract

Shikonin is a naphthoquinone isolated from the dried root of *Lithospermum erythrorhizon*, an herb used in Chinese medicine. Although several studies have indicated that shikonin exhibits antitumor activity in breast cancer, the mechanism of action remains unclear. In the present study, we performed transcriptome analysis using RNA-seq and explored the mechanism of action of shikonin in regulating the growth of different types of breast cancer cells. The IC_50_ of shikonin on MCF-7, SKBR-3 and MDA-MB-231 cells were 10.3 μΜ, 15.0 μΜ, 15.0 μΜ respectively. Our results also demonstrated that shikonin arrests the progression of cell cycle and induces apoptosis in MDA-MB-231 cells. Using RNA-seq transcriptome analysis, we found 38 common genes that significantly express in different types of breast cancer cells under shikonin treatment. In particular, our results indicated that shikonin induces the expression of dual specificity phosphatase (DUSP)-1 and DUSP2 in both RNA and protein levels. In addition, shikonin also inhibits the phosphorylation of JNK and p38, the downstream signaling molecules of DUSP1 and DUSP2. Therefore, our results suggest that shikonin induces the expression of DUSP1 and DUSP2 which consequently switches off JNK and p38 MAPK pathways and causes cell cycle arrest and apoptosis in breast cancer cells.

## Introduction

Breast cancer is one of the most common cancers and the second leading cause of cancer death among women in the United States^1^. One in eight women will be diagnosed with breast cancer in her lifetime. Approximately 70% of breast cancer patients are inoperable because of advanced tumor growth or bone metastasis^2^. Therefore, new strategies for the treatment of breast cancer are necessary. Many agents extracted from Traditional Chinese medicine (TCM) have been shown to possess anticancer activities and can be considered as alternative treatments for breast cancer^3^.

Shikonin, a naphthoquinone isolated from the Chinese herbal plant *Lithospermum erythrorhizon*, has been used to treat a variety of inflammatory and infectious diseases^4^. Several biological and pharmacological actions of shikonin have been reported, including anti-inflammatory^5^, antibacterial^6^, antiviral^7^, and antioxidant^8^ activities. In particular, shikonin has been shown to exert anticancer properties via different mechanisms on various neoplastic cells such as inducing cellular apoptosis through mitochondria-mediated pathway in human prostate cancer cells^9^, leukemia cells^10^ and gastric cancer cells^11^, inhibiting migration and metastasis in human prostate cancer cells^12^, breast cancer cells^13^ and lung cancer cells^14^, attenuating angiogenesis in murine melanoma^15^ and lung carcinoma^16^.

Transcriptome analysis associated with bioinformatics data mining tools provides an opportunity to simultaneously analyze a large number of genes/targets and identify the mechanisms of action after treatments. RNA-seq has many advantages over microarray due to it being free from the probe-specific hybridization of microarrays and has expansive coverage, allowing the unbiased detection of both coding and noncoding novel transcripts as well as low-abundance transcripts^17^.

Several breast cancer cell lines used in biological studies have been classified based on the following measures: histological type, tumour grade, lymph node status and the presence of predictive markers such as estrogen receptor (ER), progesterone receptor (PR), and human epidermal growth factor receptor 2 (HER2)^18^. Yet, some studies have provided intriguing insights into anti-breast cancer activity of shikonin^13,19–22^. Hou *et al*. indicated that shikonin inhibits cell proliferation and induces apoptosis in MCF-7 cells (Luminal A; ER^+^, PR^+/−^, HER2^−^)^20^. Zhang *et al*. demonstrated that shikonin attenuates the proliferation of both MCF-7 cells (Luminal A; ER^+^, PR^+/−^, HER2^−^) and SK-BR-3 cells (HER2; ER^−^, PR^−^, HER2^+^)^21^. In addition, Li *et al*. indicated that shikonin exhibits the cytotoxic effect in MDA-MB-231 cells (Claudin-low; ER^−^, PR^−^, HER2^−^)^22^. Although shikonin has been demonstrated as inhibiting the proliferation in different types of breast cancer cells, the mechanism of action has not been investigated. Herein, the aim of this study is to examine the mechanism of action of shikonin in regulating the growth of different types of breast cancer cells using the RNA-seq approach.

## Results

### Shikonin inhibits the growth of human breast cancer cells

**Figure 1:**
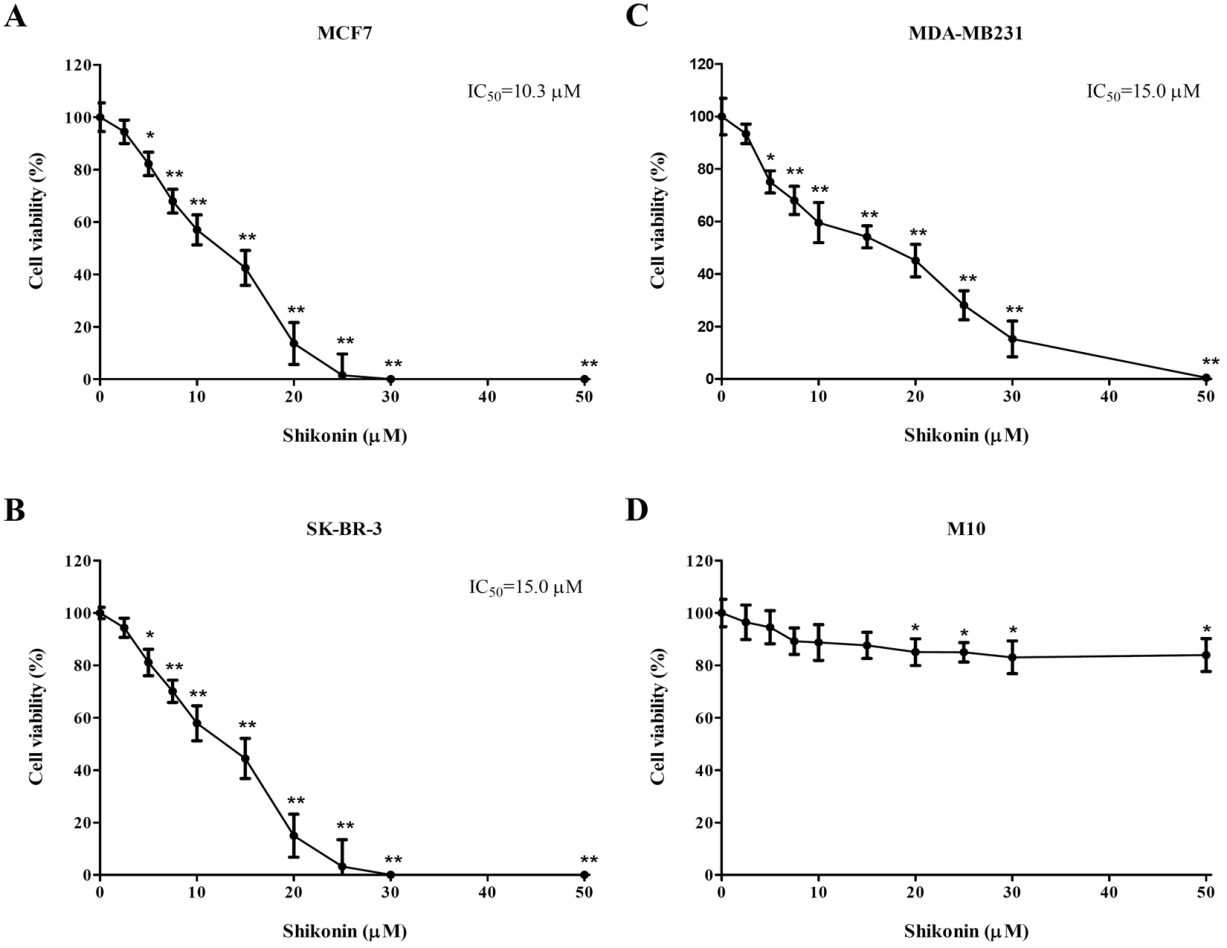
Effect of shikonin on the cell viability in breast cancer cell lines. Different breast cancer cells. (**A**) MCF-7, (**B**) SK-BR-3, (**C**) MDA-MB231, and (**D**) human mammary epithelial cells, M10, were incubated with different concentrations of shikonin (0–50 μM) for 24 h. The cell viability was determined by the MTT assay. Data points represent the mean ± SD of three independent experiments. IC_50_ values were calculated by GraphPad Prism 4.0 software using a sigmoidal curve fit based on nonlinear regression. Statistical significance was assessed by one-way ANOVA followed by Tukey post-hoc test and represented as follows: *P < 0.05 and **P < 0.01 vs. shikonin 0 μM (DMSO control).

To examine whether shikonin affects the growth of human breast cancer cells, different types of human breast cancer cell lines, MCF-7, SK-BR-3 and MDA-MB-231 were treated with shikonin for 24 hr. The cell viability was analyzed by MTT assay. As shown in Fig. 1, shikonin revealed significant cytotoxic effects in different types of human breast cancer cells in a dose-dependent manner. The IC_50_ of shikonin at 24 hr on MCF-7, SKBR-3 and MDA-MB-231 cells were 10.3 μΜ, 15.0 μΜ, 15.0 μΜ respectively (Fig. 1 A–C). In addition, we also examined whether shikonin affects the growth of human mammary epithelial cells, M10 cells. Our results indicated that human mammary epithelial cells were more resistant to shikonin-induced cytotoxicity compared to human breast cancer cells (Fig. 1D).

### Shikonin arrests the progression of cell cycle and induces apoptosis in human breast cancer cells

**Figure 2:**
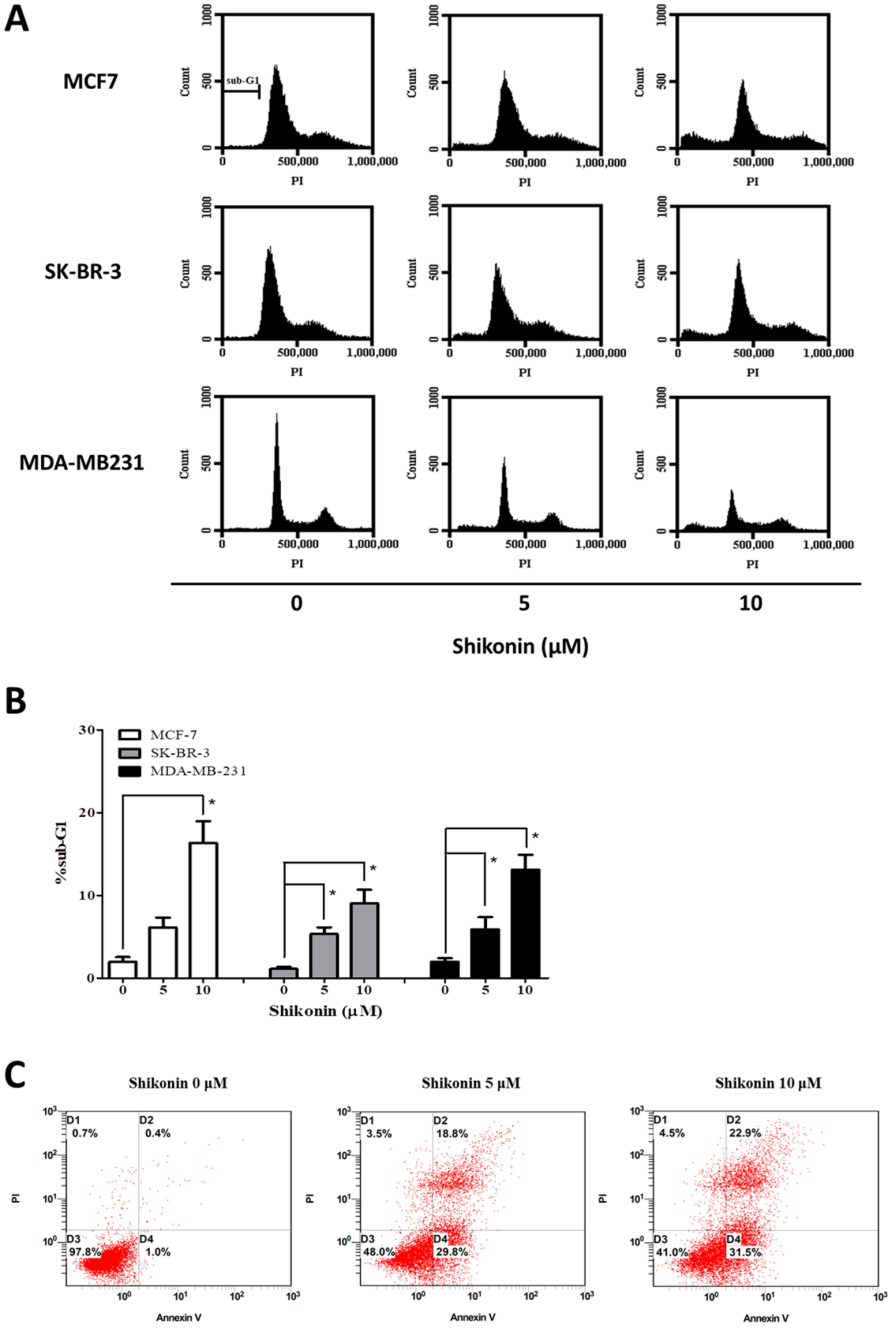
Effect of shikonin on the cell cycle progression and apoptosis in breast cancer cell lines. (**A**) Different breast cancer cells, MCF-7, SK-BR-3 and MDA-MB231, were incubated with different concentrations of shikonin (0–10 μM) for 24 h. Representative cell cycle distribution of each cell type was analyzed by flow cytometry. (**B**) Percentage of sub-G1 in different breast cancer cells under shikonin treatment was assessed by Student’s t-test. The statistical significance of the difference between two experimental measurements was represented as follows: *P < 0.05 vs. shikonin 0 μM (DMSO control). (**C**) MDA-MB231 cells were treated with different concentrations of shikonin (0–10 μM) for 24 h. Cells were collected, stained with Annexin V and PI, and analyzed by flow cytometry. Data are representative of at least three independent experiments with similar results.

To investigate the mechanisms underlying shikonin-induced inhibition of cell growth, changes in cell cycle progression of human breast cancer cells were determined after shikonin treatment using flow cytometry. As shown in Fig. 2A,B, cells in sub-G1 phase were increased under shikonin treatment in a dose-dependent manner. These results suggest that shikonin inhibited cellular proliferation of human breast cancer cell lines, MCF-7, SKBR-3 and MDA-MB-231 via arrested G1 phase of the cell cycle. Moreover, we also performed Annexin V/PI apoptosis assay. Our results showed that shikonin induced apoptosis in MDA-MB-231 cells (Fig. 2C).

### RNA-seq transcriptome analysis of different human breast cancer cell lines under shikonin treatment

**Figure 3:**
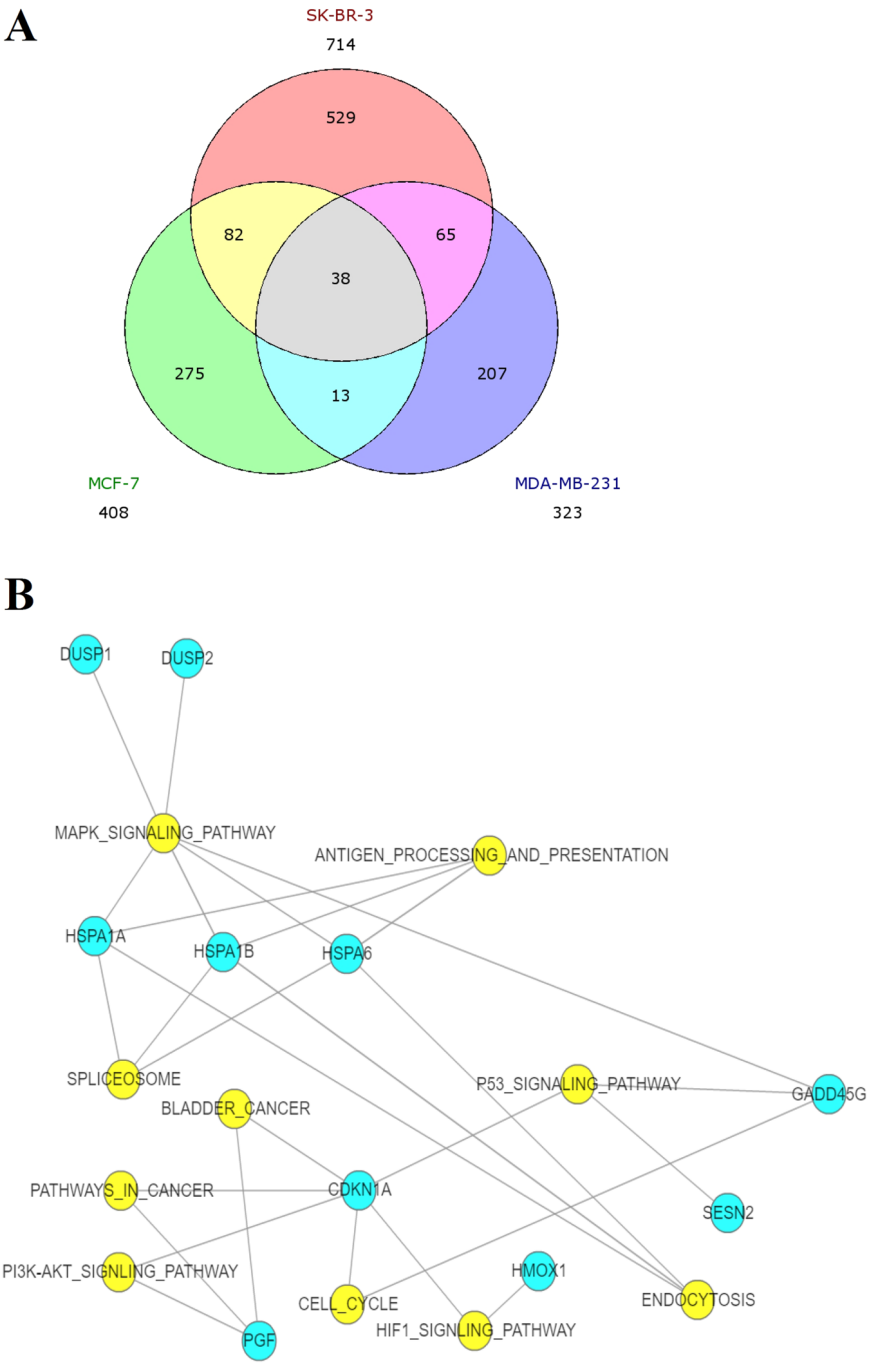
Intersectional analysis of SDE genes from breast cancer cells after shikonin treatment identified by RNA-seq and analysis of common genes using KEGG enrichment analysis. (**A**) Numbers of SDE genes from MCF-7, SK-BR-3, and MDA-MB-231 cells after shikonin treatment for 6 h were 408, 714, and 323, respectively. (**B**) 38 common genes were further used for KEGG enrichment analysis. The blue circles indicate the common genes from different types of breast cancer cells after shikonin treatment. The yellow circles show the biological functions and signaling pathway regulated by shikonin in breast cancer cells.

To study the gene expression profiling of human breast cancer cells under shikonin treatment, different human breast cancer cell lines, MCF-7, SK-BR-3 and MDA-MB-231 were treated with 10 μΜ shikonin for 6 hr. The gene expression profiling was performed using RNA-seq. As shown in Fig. 3A, numbers of significantly differentially expressed (SDE) genes (>2-fold change) in different human breast cancer cell lines under shikonin treatment were identified. The results of Venn diagrams analysis showed 38 SDE genes (termed as common genes), which were expressed in different types of breast cancer cells under shikonin treatment (Fig. 3A). Thirty-six common genes were consistently upregulated and one common gene was consistently downregulated in different types of breast cancer cells under shikonin treatment (Table 1). Only *RN7SL1* was inconsistently expressed in different types of breast cancer cells under shikonin treatment (Table 1).

**Table 1:** Differentially expressed common genes after shikonin treatment by MCF-7, SK-BR-3, and MDA-MB-231 cells. (log_2_ ratio).

Genes	Entrez ID	MCF-7	SK-BR-3	MDA-MB-231
RMRP	6023	Infinity	Infinity	Infinity
HSPA6	3310	8.48	Infinity	8.41
HMOX1	3162	6.18	5.60	2.31
PGF	5228	5.32	3.83	1.01
HSPA1A	3303	3.92	6.11	4.69
HSPA1B	3304	3.75	6.12	4.04
ATF3	467	3.69	2.03	4.08
DNAJB1	3337	3.37	4.70	3.51
OSGIN1	29948	3.19	2.79	1.36
TNFSF9	8744	3.03	2.28	3.91
PPP1R15A	23645	2.84	2.89	2.06
ARC	23237	2.70	2.84	Infinity
MIR22,MIR22HG	407004	2.64	4.00	2.09
SNAI1	6615	2.64	2.06	4.52
RN7SK	125050	2.54	1.01	Infinity
UBC	7316	2.53	2.40	1.02
IER5	51278	2.42	1.21	1.99
MAFF	23764	2.19	2.28	1.33
ZFAND2A	90637	2.12	4.07	2.99
SESN2	83667	2.00	1.28	1.22
CDKN1A	1026	1.91	3.56	2.19
HSPH1	10808	1.91	2.73	1.25
BAG3	9531	1.89	3.68	1.33
IDI2-AS1	55853	1.70	1.54	Infinity
SIK1	150094	1.61	1.00	1.30
DUSP2	1844	1.60	3.01	5.32
DUSP1	1843	1.55	2.88	1.20
GADD45G	10912	1.53	2.42	3.17
PIM1	5292	1.43	2.00	1.09
MAP1LC3B	81631	1.39	1.75	1.17
SLC25A25	114789	1.34	1.51	1.26
OSER1	51526	1.34	1.80	1.61
TSPYL2	64061	1.32	1.41	1.41
RND3	390	1.30	2.34	1.06
MAP1LC3B2	643246	1.24	1.31	1.34
CSRNP1	64651	1.06	2.25	1.79
RN7SL1	6029	−1.22	1.15	1.32
ETAA1	54465	−1.45	−1.15	−1.68

### Analysis of common genes using both functional enrichment analysis and KEGG enrichment analysis after shikonin treatment by MCF-7, SK-BR-3 and MDA-MB-231 cells

We analyzed the common genes by functional enrichment analysis. The results showed that these genes participated mainly in adjustment of cell death, apoptosis, cell cycle and cell growth (Table 2). We further analyzed the common genes by KEGG enrichment analysis. The results showed that the common SDE genes regulated by shikonin were significantly involved in MAPK signaling pathway, P53 signaling pathway, antigen processing and presentation, spliceosome, bladder cancer, endocytosis, HIF1 signaling pathway, cell cycle, pathways in cancer and the PI3K-AKT signaling pathway (Table 3). Several common genes were involved in regulating these pathways such as *HSPA1B*, *HSPA1A*, *HSPA6*, *GADD45G*, *DUSP1*, *DUSP2*, *CDKN1A*, *SESN2*, *PGF*, *HMOX1* (Table 3 and Fig. 3B).

**Table 2:** Functional enrichment analysis of common genes by GO-terms.

**Term**	**Category**	**Number of genes**	**%**	***P*** **value**	**Genes**
Response to unfolded protein	B.P.	5	1.57	1.2 × 10^−5^	HSPH1, HSPA6, HSPA1A, DNAJB1, HSPA1B, PPP1R15A
Negative regulation of apoptosis	B.P.	6	1.89	6.7 × 10^−4^	CDKN1A, HMOX1, BAG3, PIM1, UBC, HSPA1A
Negative regulation of programmed cell death	B.P.	6	1.89	7.2 × 10^−4^	CDKN1A, HMOX1, BAG3, PIM1, UBC, HSPA1A, HSPA1B
Negative regulation of cell death	B.P.	6	1.89	7.2 × 10^−4^	CDKN1A, HMOX1, BAG3, PIM1, UBC, HSPA1A, HSPA1B
Positive regulation of anti-apoptosis	B.P.	3	0.94	1.6 × 10^−3^	CDKN1A, DUSP1, HMOX1
Intracellular part	C.C.	25	7.86	3.2 × 10^−3^	HSPA1A, HSPA1B, SESN2, HSPH1, TSPYL2, SLC25A25, MAP1LC3B, HMOX1, BAG3, ETAA1, SIK1, MAFF, ARC, PIM1, MAP1LC3B2, SNAI1, RND3, CDKN1A, DUSP2, ATF3, DUSP1, CSRNP1, ZFAND2A, UBC, DNAJB1, PPP1R15A
Regulation of cell cycle	B.P.	5	1.57	4.3 × 10^−3^	CDKN1A, TSPYL2, GADD45G, PIM1, SIK1
Regulation of programmed cell death	B.P.	7	2.20	5.2 × 10^−3^	CDKN1A, DUSP1, HMOX1, BAG3, PIM1, UBC, HSPA1A, HSPA1B
Regulation of cell death	B.P.	7	2.20	5.3 × 10^−3^	CDKN1A, DUSP1, HMOX1, BAG3, PIM1, UBC, HSPA1A, HSPA1B
Regulation of transferase activity	B.P.	5	1.57	6.5 × 10^−3^	CDKN1A, TSPYL2, DUSP2, GADD45G, PIM1
Apoptosis	B.P.	6	1.89	6.8 × 10^−3^	BAG3, CSRNP1, GADD45G, UBC, TNFSF9, PPP1R15A
Intracellular membrane-bounded organelle	C.C.	21	6.60	7.3 × 10^−3^	MAFF, ARC, PIM1, HSPA1A, HSPA1B, SESN2, SNAI1, RND3, CDKN1A, TSPYL2, ATF3, DUSP2, DUSP1, SLC25A25, MAP1LC3B, HMOX1, ZFAND2A, CSRNP1, UBC, DNAJB1, SIK1, PPP1R15A
Nucleus	C.C.	16	5.03	8.2 × 10^−3^	MAFF, PIM1, HSPA1A, HSPA1B, SESN2, SNAI1, CDKN1A, TSPYL2, DUSP2, ATF3, DUSP1, HMOX1, ZFAND2A, CSRNP1, UBC, DNAJB1, SIK1
Intracellular organelle	C.C.	22	6.92	1.2 × 10^−2^	MAFF, ARC, PIM1, HSPA1A, MAP1LC3B2, HSPA1B, SESN2, SNAI1, RND3, CDKN1A, TSPYL2, ATF3, DUSP2, DUSP1, SLC25A25, MAP1LC3B, HMOX1, ZFAND2A, CSRNP1, UBC, DNAJB1, SIK1, PPP1R15A
Negative regulation of cell growth	B.P.	3	0.94	1.5 × 10^−2^	CDKN1A, TSPYL2, OSGIN1
Negative regulation of cellular process	B.P.	9	2.83	1.5 × 10^−2^	CDKN1A, TSPYL2, HMOX1, BAG3, PIM1, UBC, OSGIN1, HSPA1A, HSPA1B, SIK1
Regulation of phosphorus metabolic process	B.P.	5	1.57	1.6 × 10^−2^	CDKN1A, TSPYL2, DUSP2, GADD45G, PIM1
Negative regulation of cell size	B.P.	3	0.94	1.7 × 10^−2^	CDKN1A, TSPYL2, OSGIN1
Negative regulation of catalytic activity	B.P.	4	1.26	1.8 × 10^−2^	CDKN1A, DUSP2, GADD45G, UBC
Cell cycle arrest	B.P.	3	0.94	1.8 × 10^−2^	CDKN1A, SESN2, PPP1R15A
Cytoplasm	C.C.	19	5.97	2.1 × 10^−2^	ARC, PIM1, HSPA1A, MAP1LC3B2, HSPA1B, SESN2, RND3, HSPH1, CDKN1A, TSPYL2, SLC25A25, MAP1LC3B, HMOX1, ZFAND2A, BAG3, UBC, ETAA1, DNAJB1, SIK1, PPP1R15A
Negative regulation of growth	B.P.	3	0.94	2.1 × 10^−2^	CDKN1A, TSPYL2, OSGIN1
Cellular protein metabolic process	B.P.	10	3.14	3.9 × 10^−2^	DUSP2, DUSP1, MAP1LC3B, BAG3, GADD45G, PIM1, UBC, MAP1LC3B2, DNAJB1, SIK1

B.P., biological process; C.C., cellular component.

**Table 3:** KEGG enrichment analysis of common genes after shikonin treatment by MCF-7, SK-BR-3 and MDA-MB-231 cells.

Pathway	*P* value (−log_10_)	Genes
MAPK signaling pathway	5.96	HSPA1B,HSPA1A,HSPA6,GADD45G,DUSP1,DUSP2
P53 signaling pathway	3.98	CDKN1A,GADD45G,SESN2
Antigen processing and presentation	3.67	HSPA1B,HSPA1A,HSPA6
Spliceosome	3.22	HSPA1B,HSPA1A,HSPA6
Bladder cancer	2.85	CDKN1A,PGF
Endocytosis	2.78	HSPA1B,HSPA1A,HSPA6
HIF1 signaling pathway	2.09	CDKN1A,HMOX1
Cell cycle	1.93	CDKN1A,GADD45G
Pathways in cancer	1.19	CDKN1A,PGF
PI3K-AKT signaling pathway	1.15	CDKN1A,PGF

### Validation of RNA-seq data by qRT-PCR

**Figure 4:**
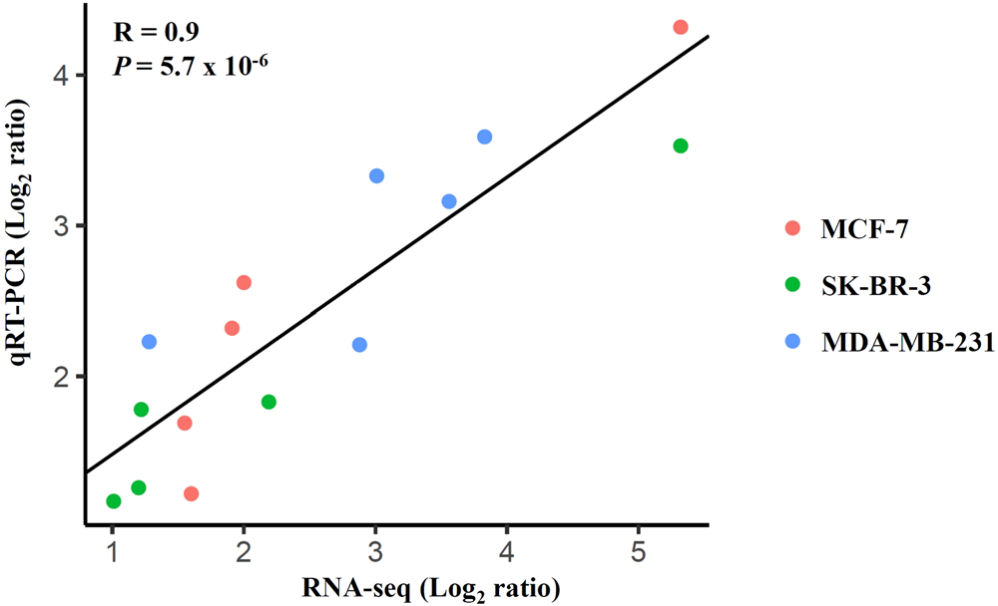
Correlation of gene expression ratios between RNA-seq and qRT-PCR. A total of 15 RNA-seq samples were validated by qRT-PCR (5 representative genes, *DUSP1*, *DUSP2*, *CDKN1A*, *SESN2*, and *PGF*, in three different types of breast human breast cancer cells. Data from both RNA-seq and qRT-PCR were normalized by setting the expression level of untreated control.

To further validate the results of RNA-seq, qRT-PCR was performed on 5 genes (*DUSP1*, *DUSP2*, *CDKN1A*, *SESN2*, *PGF*) randomly selected from common genes using the same RNA samples that were used in RNA-seq. A total of 15 RNA-seq samples were validated by qRT-PCR (5 representative genes in three different types of breast human breast cancer cells. Correlation of the expression ratios from the RNA-seq and qRT-PCR data were highly correlated (R = 0.9; *P* = 5.7 × 10^−6^) (Fig. 4).

### Shikonin enhances the expression of DUSP1 and DUSP2 in both RNA and protein levels and decreases the phosphorylation of JNK and p38

**Figure 5:**
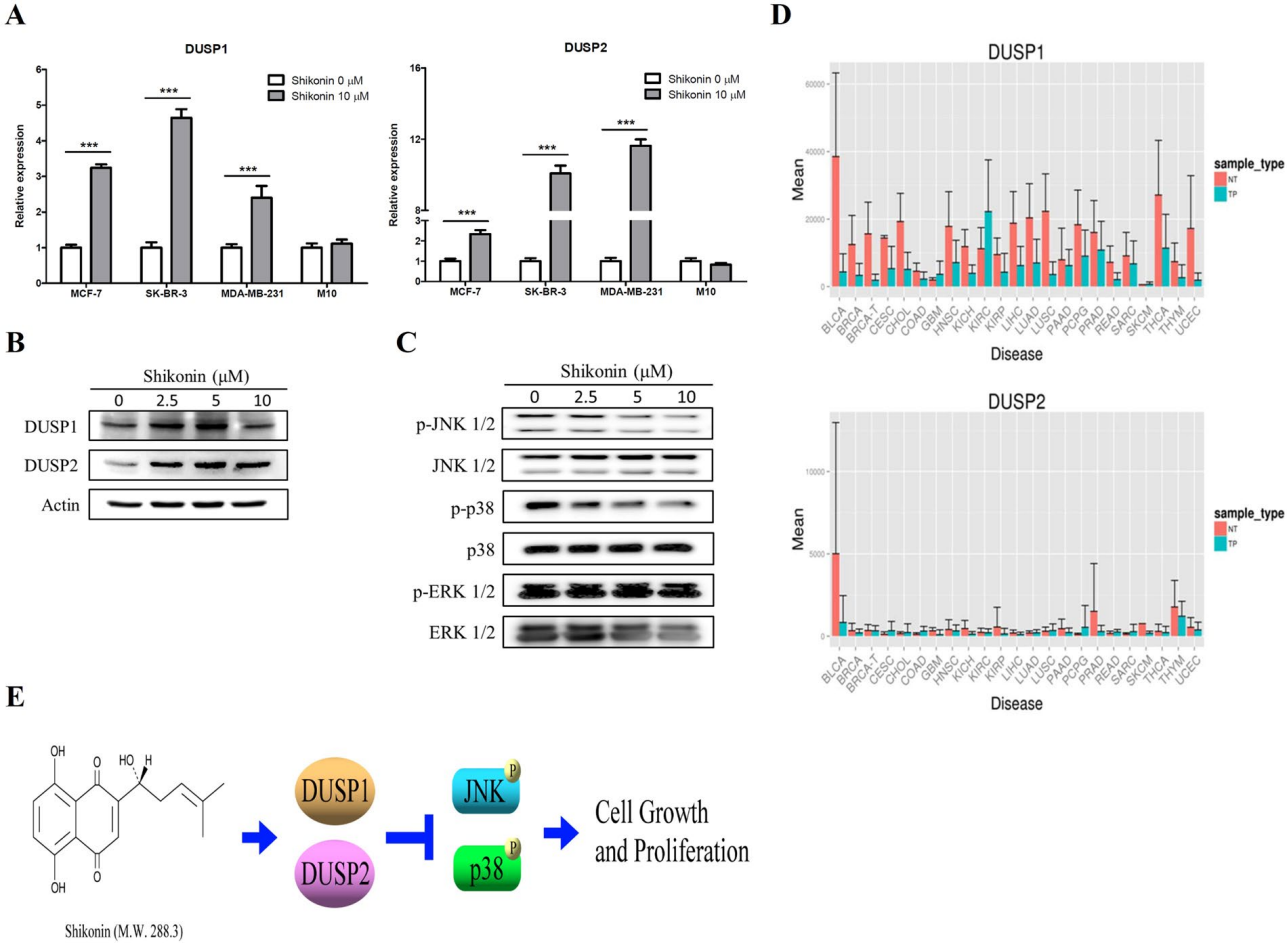
Effect of shikonin on the expression level of DUSP1 and DUSP2 and the activation of MAPKs pathway in breast cancer cells. (**A**) Different breast cancer cells, MCF-7, SK-BR-3 and MDA-MB231, and human mammary epithelial cells, M10, were incubated with or without shikonin 10 μM for 6 h. The expressions of *DUSP1* and *DUSP2* were determined by qRT-PCR. Data are presented as mean ± SD from three independent experiments. The statistical significance of the difference between two experimental measurements was assessed by Student’s t-test and represented as follows: ***P < 0.001 vs. shikonin 0 μM (DMSO control). (**B**) MB-231 cells were treated with different concentrations of shikonin for 6 hr. The expressions of DUSP1 and DUSP2 were analyzed by Western blot. The expression of β-actin was used as a loading control. (**C**) MB-231 cells were treated with different concentrations of shikonin for 24 hr. The expressions of phospho-JNK 1/2, JNK 1/2, phospho-p38, p38, phospho-ERK1/2, and ERK 1/2 were analyzed by Western blot. The Western blotting results are representative of results obtained in three separate experiments. (**D**) Gene expression levels of DUSP1 and DUSP2 from TCGA RNA-seq data in many types of cancer were analyzed by DriverDB^23,24^. The list of abbreviations is shown as follows: BLCA: bladder urothelial carcinoma; BRCA: breast invasive carcinoma; BRCA-T: BRCA-associated triple-negative breast cancer; CESC: cervical squamous cell carcinoma and endocervical adenocarcinoma; CHOL: COAD: colon adenocarcinoma; GBM: glioblastoma multiforme; HNSC: head-neck squamous cell carcinoma; KIRC: kidney renal clear cell carcinoma; KIRP: kidney renal papillary cell carcinoma; LIHC: liver hepatocellular carcinoma; LUAD: lung adenocarcinoma; LUSC: lung squamous cell carcinoma; PAAD: pancreatic adenocarcinoma; PCPG: pheochromocytoma and paraganglioma; PRAD: prostate adenocarcinoma; READ: rectum adenocarcinoma; SARC: sarcoma; SKCM: skin cutaneous melanoma; THCA: thyroid cancer; THYM: thymoma; UCEC: uterine corpus endometrial carcinoma. (**E**) Shikonin inhibits cell growth and induces apoptosis in different types of breast cancer cells through enhances the expression of DUSP1 and DUSP2 and reduces the activity of their downstream signaling molecules, JNK and p38.

The results of RNA-seq showed that shikonin induces the expression of *DUSP1* and *DUSP2* in breast cancer cells (Table 1). We confirmed the results of RNA-seq using qRT-PCR. As shown in Fig. 5A, the expression of *DUSP1* and *DUSP2* was increased in MCF-7, SK-BR-3 and MDA-MB-231 cells after shikonin treatment. However, there was no effect on the expression of *DUSP1* and *DUSP2* in M10 cells after shikonin treatment. In addition, we examined the expression of DUSP1 and DUSP2 in MDA-MB-231 after shikonin treatment. As shown in Fig. 5B, shikonin induced the expression of DUSP1 and DUSP2 in MDA-MB-231 cells. Furthermore, our results also showed that shikonin decreased the phosphorylation of JNK 1/2 and p38 in MDA-MB-231 cells, whereas the phosphorylation of ERK 1/2 exhibited no effect after shikonin treatment in MDA-MB-231 cells (Fig. 5C). On the other hand, we analyzed the expression of DUSP1 and DUSP2 using DriverDB^23,24^. As shown in Fig. 5D, DUSP1 and DUSP2 were down-regulated in several types of cancers.

## Discussion

The use of Chinese herbal medicine for health promotion and adjuvant therapy is becoming increasingly popular worldwide. Zicao, the dried root of *Lithospermum erythrorhizon*, is a Chinese herbal medicine widely used for its anti-inflammatory properties in China, Japan, Korea, etc.^25^. Shikonin is a major component of zicao and has been reported to suppress the growth of several types of cancer through a wide spectrum of anticancer mechanisms^4^. However, the mechanism of action of shikonin in regulating the growth of breast cancer cells is limited.

Various subtypes of breast cancer have distinct prevalence and outcomes. In the present study, we used three different types of breast cancer cell lines, MCF-7 (Luminal A; ER^+^, PR^+/−^, HER2^−^, good outcome), SK-BR-3 (HER2; ER^−^, PR^−^, HER2^+^, poor outcome), MDA-MB-231 (Claudin-low; ER^−^, PR^−^, HER2^−^, poor outcome)^26^ and demonstrated that shikonin inhibits the growth of these cancer cells including arresting the progression of the cell cycle and inducing apoptosis. To further explore the mechanism of action of shikonin in regulating the growth of different types of breast cancer cells, we analyzed the gene expression profiling of different types of breast cancer cells using RNA-seq. We found 38 common genes regulated by shikonin in different types of breast cancer cells and further analyzed these common genes using KEGG enrichment analysis. The analytic results indicated that these common genes were significantly involved in the MAPK signaling pathway, P53 signaling pathway, antigen processing and presentation, spliceosome, bladder cancer, endocytosis, HIF1 signaling pathway, cell cycle, pathways in cancer and the PI3K-AKT signaling pathway.

In particular, the results of RNA-seq pointed out that shikonin induced the expression of both *DUSP1* and *DUSP2*, the upstream regulators of MAPK signaling pathway. Also, the results of qRT-PCR confirmed that shikonin induced the expression of both *DUSP1* and *DUSP2* in different types of breast cancer cells. The expression ratios from RNA-seq and qRT-PCR data were highly correlated. Moreover, our experimental results also demonstrated that shikonin induced the protein expression of both DUSP1 and DUSP2 in different types of breast cancer cells. In addition, we also found that DUSP1 and DUSP2 were down-regulated in several types of cancers. Therefore, induction of DUSP1 and DUSP2 might be a therapeutic strategy for treating cancer.

DUSP1 and DUSP2 are the members of the threonine-tyrosine dual-specificity phosphatase family which play an important role in regulating the dephosphorylation of threonine and tyrosine residues on MAPKs^27^. MAPKs are signaling components that link extracellular signals to regulate a wide range of cellular processes in cancer cells including growth, differentiation, migration and apoptosis^28^. Our experimental results indicated that shikonin reduced the phosphorylation of JNK 1/2 and P38 in MDA-MB-231 cells. Previous studies pointed out that JNK and P38 MAPK pathways regulated the progression of cell cycle, modulated the cell survival and differentiation, and controled the balance of apoptosis and autophagy in response to chemotherapeutic agents in cancer cells^29,30^. Therefore, we suggest that shikonin induces the expression of DUSP1 and DUSP2 which consequently switches off JNK and p38 MAPK pathways and causes cell cycle arrest and apoptosis in breast cancer cells.

In summary, our results showed that shikonin inhibits cell growth and induces apoptosis in different types of breast cancer cells. We further examined the transcriptome regulation of shikonin in different types of breast cancer cells using the RNA-seq. We firstly reported that shikonin affects the expression of common genes among different types of breast cancer cells and is involved in regulating several anticancer mechanisms of action. Particularly, our results indicated that shikonin induces the expression DUSP1 and DUSP2 and reduces the activity of their downstream signaling molecules, JNK and p38. These results suggest that shikonin induces apoptosis through enhancing the expression of DUSP1 and DUSP2 (Fig. 5E).

## Materials and Methods

### Chemicals and reagents

Cell culture medium, Dulbecoo’s modified Eagle’s medium (DMEM), DMEM/F12, alpha-Minimum essential medium, trypsin, penicillin–streptomycin, and Dulbecco’s Phosphate Buffered Saline (DPBS) were purchased from Corning Cellgro (Manassas, VA, USA). Fetal bovine serum (FBS) was purchased from Gibco (Invitrogen, Carlsbad, CA, USA). Purified shikonin (≥98%), dimethyl sulfoxide (DMSO), 3-(4,5-Dimethylthiazol-2-yl)-2,5-diphenyltetrazolium bromide (MTT), hydrochloric acid (HCl), isopropanol, RIPA buffer, protease inhibitor cocktail and Tris-buffered saline/Tween 20 (TBST) were purchased from Sigma (St. Louis, MO, USA). Antibodies against dual specificity phosphatase (DUSP)-1, DUSP-2, β-actin and horseradish peroxidase (HRP)-conjugated secondary antibodies were purchased from Santa Cruz Biotechnology (Dallas, TX, USA). Antibodies against mouse phospho-JNK 1/2, JNK 1/2, phospho-p38 mitogen-activated protein kinase (MAPK), and p38 MAPK, and phospho-ERK 1/2, ERK 1/2 were purchased from Cell Signaling (Farmingdale, NY, USA). Pierce BCA Protein Assay Kit and ECL chemiluminescence substrate were purchased from Thermo Scientific (Rockford, IL, USA). TRIzol reagent, SuperScript® VILO™ cDNA Synthesis kit, SYBR® GreenER™ qPCR SuperMixes were purchased from Life Technologies (Carlsbad, CA, USA). RNA 6000 Nano LabChip kit was obtained from Agilent Technologies (Palo Alto, CA, USA).

### Cell culture

Human breast cancer cell lines, MCF-7, SK-BR-3, and MDA-MB-231 cells were obtained from American Type Culture Collection (Manassas, VA, USA). Human mammary epithelial cell line, M10, was purchased from Bioresource Collection and Research Center (Hsinchu, Taiwan). MCF-7 and MDA-MB-231 cells were maintained in DMEM supplemented with 10% heat-inactivated FBS and 100 μg/mL of penicillin-streptomycin, SK-BR-3 cells were maintained in DMEM/F12 supplemented with 10% heat-inactivated FBS and 100 μg/mL of penicillin-streptomycin, and M10 cells were maintained in alpha-Minimum essential medium supplemented with 10% heat-inactivated FBS and 100 μg/mL of penicillin-streptomycin. All cell lines were cultured in a humidified atmosphere with 5% CO_2_ at 37 °C. Shikonin was dissolved in DMSO. All treatments were adjusted to equal concentrations of DMSO between 0.1~0.2%.

### Cell viability assay

Cell viability was determined using the MTT colorimetric assay. Cells were seeded in 96-well plates in culture medium at density of 1 × 10^4^ cells/well overnight and then treated with various concentrations of shikonin between 0~50 μM for 24 hr. Subsequently, MTT in DPBS (0.1 mg) was added to each well and incubated for 4 hours at 37 °C. The MTT formazan crystals were dissolved with the addition of acid–isopropanol (1 portion of 4 N HCl: 100 portion of isopropanol). After 20 min, the optical density (OD) was measured with a microplate reader (BIO-RAD, Hercules, CA, USA) at 570 nm.

### Cell cycle analysis

MCF-7, SK-BR-3, and MDA-MB-231 cells were treated with different doses of shikonin (0, 5, and 10 μM) for 16 hr. Cells were trypsinized, washed twice by cold PBS, and fixed in 70% cold ethanol overnight at −20 °C. After fixation, cells were washed twice by cold PBS and stained with PI/Triton X-100 staining solution (0.1% Triton X-100, 2 mg/mL PI, and 0.2 mg/mL DNase-free RNase) for 30 minutes. Samples were analyzed using a flow cytometry FC500 (Beckman Coulter, Krefeld, Germany).

### Apoptosis assay

MDA-MB-231 cells were treated with different doses of shikonin (0, 5, and 10 μM) for 24 hr. Cells were trypsinized, washed twice by cold PBS, and stained with Alexa Fluor® 488 Annexin V and propidium iodide (PI) according to manufacturer’s protocol (Thermo Fisher Scientific, Rockford, IL, USA). Apoptotic cells were determined using FC500 flow cytometer (Beckman-Coulter, Fullerton, CA, USA). Ten thousand events were collected per sample. Data were analyzed by CXP analysis software (Beckman-Coulter, Fullerton, CA, USA).

### RNA preparation and RNA-seq

Total RNA was extracted with TRIzol reagent following the recommendations of the manufacturer. The quality of total RNA was evaluated using the Agilent 2100 bioanalyzer (Agilent, Palo Alto, CA, USA) with the RNA 6000 Nano LabChip kit. RNA-seq libraries were prepared Illumina® TruSeq RNA Library Prep Kit v2 and were sequenced using Illumina® Hiseq2500 to obtain 150-bp paired-end reads. The sequencing depth for each sample was >20 million reads. The reads were aligned with TopHat 2.0.13 to GRCh37 with default parameters, and then were assembled by Cufflink 2.2.1, using Ensembl v75 annotations. Transcript abundance was measured in fragments per kb of exon per million fragments mapped (FPKM). The RNA-seq data is available at GEO (GSE100687).

### Quantitative real-time PCR (qRT-PCR)

cDNA was synthesized from 1 to 2 μg RNA using SuperScript® VILO™ cDNA Synthesis kit according to the manufacturer’s instructions (Life Technologies, Carlsbad, CA, USA). The PCR reaction was performed by iCycler thermal cycler (Bio-Rad, Hercules, CA, USA) using SYBR® GreenER™ qPCR SuperMixes with PCR primers (CDKN1A: F-TGTCCGTCAGAACCCATGC, R-AAAGTCGAAGTTCCATCGCTC; DUSP1: F-AGTACCCCACTCTACGATCAGG, R-GAAGCGTGATACGCACTGC; DUSP2: F-GGGCTCCTGTCTACGACCA, R-GCAGGTCTGACGAGTGACTG; PGF: F-GAACGGCTCGTCAGAGGTG, R-ACAGTGCAGATTCTCATCGCC; SESN2: F-AAGGACTACCTGCGGTTCG, R-CGCCCAGAGGACATCAGTG; 18S rRNA: F-GGAATTGACGGAAGGGCACCACC, R-GTGCAGCCCCGGACATCTAAGG). The relative level of target genes from each sample was determined by normalizing to 18S rRNA. All experiments were repeated at least twice to duplicate results.

### Functional enrichment analysis

For the differentially expressed genes associated with treatment responses, we performed functional enrichment analysis, as described in our previous studies^23,24^ to interpret their biological functions. In brief, we used the topGO and GeneAnswers packages of Bioconductor to calculate the topology of the GO graph, as well as to visualize the many-to-many relationships between GO terms and genes. In the “Pathway” analysis, we used collections from KEGG^31^, PID^32^, Biocarta (http://www.biocarta.com/), REACTOME^33^, and MSigDB^34^ to annotate driver genes.

### Statistical analysis

Data were obtained from at least three independent experiments and expressed as the mean ± standard deviation for each group. Statistical analyses, including Student’s t-test, one-way analysis of variance and regression analysis were performed using GraphPad Prism 4.0 software (GraphPad, Inc., La Jolla, CA, USA). *P* < 0.05 was considered to indicate a statistically significant difference.

## Electronic supplementary material

Raw data of Western blot
