# Supplementary material for: RNA-seq transcriptome analysis of breast cancer cell lines under shikonin treatment
Source: Sci Rep. 2018 Feb 8;8:2672. doi: 10.1038/s41598-018-21065-x (PMC5805692; doi:10.1038/s41598-018-21065-x)
Supplement: Supplementary file 1 — Raw data of Western blot [file 41598_2018_21065_MOESM1_ESM.docx]

**RNA-seq transcriptome analysis of breast cancer cell lines under shikonin treatment**

Kuo-Hua Lin^1, #^, Ming-Yii Huang^2, #^, Wei-Chung Cheng^3^, Shu-Chi Wang^4^, Shih-Hua Fang^5^, Hung-Pin Tu^6^, Chia-Cheng Su^7, 8, 9^, Yung-Li Hung^10^, Po-Len Liu^11^, Chi-Shuo Chen^12^, Yu-Ting Wang^8^, Chia-Yang Li^8, 13, 14, *^

^1^ Department of Surgery, Changhua Christian Hospital, Changhua City 50006, Taiwan.

^2^ Department of Radiation Oncology, Cancer Center, Kaohsiung Medical University Hospital, Kaohsiung Medical University, Kaohsiung 80708, Taiwan.

^3^ Graduate Institute of Biomedical Sciences, China Medical University, Taichung, 40402, Taiwan.

^4^ Health Management Center, Kaohsiung Medical University Hospital, Kaohsiung Medical University, Kaohsiung 80708, Taiwan.

^5^ Institute of Athletics, National Taiwan University of Sport, Taichung 40404, Taiwan.

^6^ Department of Public Health and Environmental Medicine, School of Medicine, College of Medicine, Kaohsiung Medical University, Kaohsiung 80708, Taiwan.

^7^ Division of Urology, Department of Surgery, Chi-Mei Medical Center, Tainan 71004, Taiwan.

^8^ Graduate Institute of Medicine, College of Medicine, Kaohsiung Medical University,

Kaohsiung 80708, Taiwan.

^9^ Department of Senior Citizen Service Management, Chia Nan University of Pharmacy and Science, Tainan 71710, Taiwan.

^10^ Graduate School of Sport Sciences, Waseda University, Tokorozawa 359-1192, Japan.

^11^ Department of Respiratory Therapy, College of Medicine, Kaohsiung Medical University, Kaohsiung 80708, Taiwan.

^12^ Department of Biomedical Engineering and Environmental Sciences, National Tsing Hua University, Hsinchu 30013, Taiwan.

^13^ Center for Infectious Disease and Cancer Research, Kaohsiung Medical University, Kaohsiung 80708, Taiwan.

^14^ Department of Medical Research, Kaohsiung Medical University Hospital, Kaohsiung, Kaohsiung 80756, Taiwan

^#^These authors contributed equally to this work.

Correspondence and requests for materials should be addressed to C.-Y.L. (email: [chiayangli@kmu.edu.tw](mailto:chiayangli@kmu.edu.tw))
